# The Study of *IL-10* and *IL-17A* Genes Expression in Patients with Different Stages of Asthma: a Case-Control Study

**Published:** 2018-03

**Authors:** Elham Zonoobi, Kayvan Saeedfar, Guitti Pourdowlat, Mohammad Reza Masjedi, Mehrdad Behmanesh

**Affiliations:** 1 Department of Genetics, Faculty of Biological Sciences, Tarbiat Modares University, Tehran, Iran; 2 Tracheal Diseases Research Center, National Research Institute of Tuberculosis and Lung Diseases, Shahid Beheshti University of Medical Sciences, Tehran, Iran; 3 Chronic Respiratory Diseases Research Center (CRDRC), National Research Institute of Tuberculosis and Lung Diseases (NRITLD), Shahid Beheshti University of Medical Sciences, Tehran, Iran; 4 Tobacco Control Research Center, Iranian Anti-Tobacco Association, Tehran, Iran

**Keywords:** Asthma, Cytokine expression, IL-17A, IL-10, PBMC, qPCR

## Abstract

**Background:**

Asthma is considered as a complex disorder in which genetics and environment play crucial role in its susceptibility. In addition to the huge financial costs that significantly reduce the quality of life of the patients and their families, it causes high prevalence of lung diseases. Finding contributing new genetic factors involved in early diagnosis or progression of asthma can provide novel approaches for treatment or managing of asthma. In the present study, the potential role of two key cytokines of *IL-10* and *IL-17A* was investigated in asthma pathogenesis.

**Materials and Methods:**

Using real-time PCR technique, we analyzed the expression levels of target genes in two groups of mild and severe asthma patient in comparison with healthy individuals.

**Results:**

In comparison with control population, obtained data showed 4 and 7-fold down-regulation of *IL-17A* in the group of mild and severe asthma, respectively. Down-regulation of *IL-17A* showed a significant correlation with progression of asthma severity. While *IL-10* showed up to 10-fold down-regulation in the group of severe asthma, its expression level was not correlated with severity of asthma.

**Conclusion:**

Obtained data revealed that deregulation *IL-10* and *IL-17A* have potential to play crucial role in pathogenesis and prognosis of asthma. Observed down-regulation of these cytokines in blood cells suggests their usefulness as a marker in diagnosis of asthmatic types in patients.

## INTRODUCTION

Asthma is, a common complex disease, characterized by chronic inflammation of airway, variable airflow limitation, repetitive cough, wheezing, shortness of breath and chest tightness (1). Asthma is associated with considerable morbidity, avoidable mortality and substantial costs to society (2). According to the estimation of World Health Organization (WHO) more than 300 million people currently suffer from asthma worldwide and this number is expected to grow to 400 million by 2025 (3).

The definition and classification of severe asthma, which afflicts only small percentage of asthma population (<5–10%), has been under debate since past decades. An international consensus statement and a unique definition, classification and diagnostic algorithm of Severe Refractory Asthma (SRA) was published by the Innovative Medicine Initiative (IMI) in 2011. According to this statement “the term severe refractory asthma should be reversed for patients with asthma in whom other diagnoses have been excluded, aggravating comorbidities have been treated, environmental exacerbating factors have been eliminated (if possible) and also compliance with appropriate treatment has been checked, but still have poor asthma control or frequent (≥2) intense exacerbations during a year despite the prescription of high-severity treatment or only adequate control can be maintained when taking systemic corticosteroids and are thereby at risk of serious side effects of treatment”(4).

Although environmental factors play key role in the pathogenesis of asthma, there are multiple reported genes which confer susceptibility to this disease (5,6). Owing to complexity, it has been difficult to identify the genetic basis of such complex genetic disorders and yet, the genetic contribution of asthma disease is remained to be investigated (7). Accumulating evidence suggest that immune system cells such as TH17 cells and their related cytokines are involved in the pathophysiology of asthma (8,9). These data emphasize the prominent role of cytokines and their receptors in promotion of allergy and asthma (10–12).

IL-10 is a cytokine derived from CD4^+^ T-helper type 2 (TH2) cells (13,14) identified as a suppressor of cytokines from T-helper type 1 (TH1) cells (15,16). IL-10 contributes in the pathophysiologic mechanism of inflammatory disease since it has been shown to regulate both cellular and humoral immunity (17).

Distinct responses of IL-17-producing cells in inflammatory conditions (18) together with its increase in lung lesions (19,20), highlighted the potential involvement of IL-17 in asthma (21) and potentiality it could be a modifier gene specific to asthma. In addition, IL-17 family members including IL-17A, IL-17B, IL-17C, IL-17D, IL-17E, and IL-17F (22,23) are known to be important regulator of neutrophilic inflammation (24) and aberration in their production may drive severe forms of the disease (21,25). Among *IL-17* family members, the expression patterns and function of *IL-17A* is not well understood.

In this study, we evaluated the expression alteration of Interleukin-10 (*IL-10*) and Interleukin-17A (*IL-*17A) in some categories of asthma patients with different level of severity versus healthy controls.

## MATERIALS AND METHODS

### Patients and control

This cross-sectional study is done based on a shared research by Tarbiat Modares University (TMU) and the National Research Institute of Tuberculosis and Lung Diseases (NRITLD) of Tehran-Iran. The study included a population of asthma patients who referred to NRITLD center and was conducted with 13 patients with SRA, 14 non-severe asthma and 26 healthy controls from 2012–14. Patients were enrolled from the same clinic using criteria outlined by the Global Initiative for Asthma (GINA) “Global Strategy for Asthma Management and Prevention: Global Initiative for Asthma (GINA); 2012. Available from: Http://www.ginasthma.org/.”) and 2011 international consensus for definition of SRA (Bel et al.). Patient involvement was approved by certified pulmonologists. Healthy participants had no history of any compounding disorders and history of hospitalization at the time study. All individuals enrolled voluntary and written informed consent was obtained prior to the blood sampling. The demographic and clinical data of patients were kept confidential and there was not any intervention applied throughout their clinical management. The local research ethic committee of NRITLD approved all stages of the research under the code sbmu1.REC.1391.1. Demographics of subjects are summarized in table 1.

**Table 1.** Demographic characteristics of the participants

	**Severe Asthma**	**Asthma**	**Healthy**	**Total**	**P-value**
**Gender**					
Male	8	9	23	40	
Female	5	5	3	13	
Total	13	14	26	53	
**Age**					
Mean (SE)	50.23 (3.80)	54.71 (4.31)	43(1.52)	49.32(3.21)	0.01
%95 CI	41.95–58.51	45.39–64.4	40.02–45.98	44.44–54.31	
Min–Max	21–76	24–80	28–65	21–80	
**BMI**					
Mean (SE)	26.39 (1.25)	26.41 (0.81)	26.48(0.8)	26.43(0.96)	0.99
%95 CI	23.65–29.14	24.65–28.16	24.89–28.06	24.39–28.46	
Min–Max	19.53–35.49	20.05–30.12	18.64–34.09	18.64–35.49	

### RNA preparation of Peripheral Blood Mononuclear Cells

Three milliliters of whole blood were taken and collected in the anti-coagulant EDTA tubes from each participant. Peripheral Blood Mononuclear Cells (PBMCs) were isolated by density gradient centrifugation on Ficoll-Paque solution (lympholyte, Cedarlane, Sweden) according to manufacturer's instructions as described.

Total RNA was extracted from PBMCs by RNX^™^-Plus reagent (SinaClon, Iran) based on the manufacturer's instructions. Isolated RNAs were treated with DNase I (Fermentas, Lithuania) for 15 min at 37° C to remove any genomic DNA contamination. RNAs quality and quantity were verified by 1% agarose gel electrophoresis and spectrophotometry, respectively.

### cDNA synthesis and Genes expression analysis

Reverse transcription of RNA was performed using the RevertAid^™^ M-MuLV RT (Fermentas, Lithuania) based on manufacturer's instructions, with Oligo dT and Random Hexamer primers (MWG, Germany). Generated cDNA was used for subsequent gene expression analysis by Real-time PCR technique. Quantitative genes expression analysis was performed using real time PCR standard method by Applied Biosystems Step One PCR System (Applied Biosystem/MDS SCIEX, Foster City, CA, USA).

To examine genes expression of interleukins 17A (*IL-17A*) and 10 (*IL-10*) specific primers were designed by Oligo analyzer software (version 7). The relative expression of IL-17AmRNA:

(Forward: CTTCCCCCGGACTGTGATGGTCAA Reverse: TCATGTGGTAGTCCACGTTCCCAT), IL-10: (Forward: CCCAGACATCAAGGCGCATGTG Reverse: GTAGATGCCTTTCTCTTGGAGC) were normalized to Glyceraldehyde 3-phosphate dehydrogenase (*GAPDH*): (Forward: CCATGAGAAGTATGACAAC Reverse: GAGTCCTTCCACGATACC) which was used as the internal control.

PCR was carried out in final reaction volume of 20 μl containing 4 pM of each forward and reverse primers, 10 ng of cDNA template, and 4 μl of 5X EvaGreen^®^ qPCR Mix Plus (ROX) (Solis BioDyne, Estonia). The thermal reaction condition was as follows: initial denaturation at 95 °C for 10 min, followed by 40 cycles of denaturation at 95 °C for 15 sec, annealing at 60 °C for 20 sec, and extension at 72 °C for 20 sec. All of the samples were tested in triplicate and the normalized expression was used for data analysis and the specificity of qPCR reactions was verified by a single band after 12% polyacrylamide gel electrophoresis. The ΔCt values were determined by subtracting the average of *GAPDH* Ct value from the average *IL-17A*or *IL-10*, Ct value to calculate the normalized expression. Relative expression was calculated by 2^−ΔΔCt^ formula as described (Livak).

### Data analysis

All statistical analyses were performed using the Student's t-test with SPSS software version 18.0 (SPSS, Inc, Chicago, IL, USA) and GraphPad Prism version 6.0 (GraphPad Prism Software, Inc., San Diego, CA). P-values less than 0.05 were considered as statistically significant, and all statistical tests were two-tailed.

## RESULTS

### Transcription analysis of IL-10 and IL-17A in mild and severe asthma patient compared with normal control population

**Figure 1.**
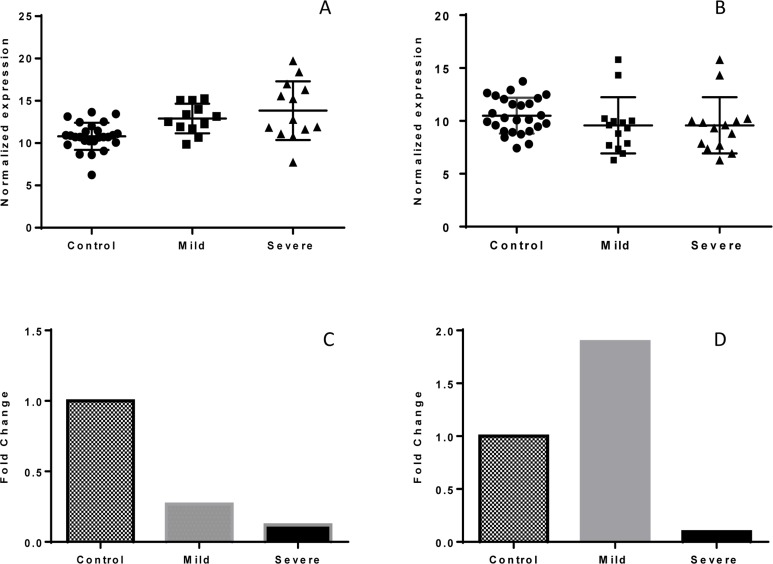
Comparison of normalized and fold change expression of *IL-17A*and *IL*10 in control, mild and severe asthma. The normalized expression of *IL-17A* is statistically different between severe asthma and control group (P value = 0.006) (A). While *IL-17A* is down-regulated in both mild and severe asthma samples, *IL-10* down-regulation is limited to severe asthma (B). The fold change analysis of gene expression in *IL-17A* showed a significant decrease of 3.8 and 8.2-times in mild and severe asthma in comparison to the control, respectively (C). This analysis for *IL-10* gene expression showed a diverse pattern in mild (about 2-times) increasing and in severe asthma (about 10 –times) decreasing in comparison to the control group (D).

Using real time PCR technique expression analysis of *IL-10* and *IL-17A* genes was performed. Data showed that while, the normalized expression level of *IL-17A* underwent a down-regulation in both mild (P=0.332) and severe asthma (P=0.006) affected samples, only in severe asthma the expression was statistically significant in comparison with control group (Figure 1A).

The normalized expression of *IL10* showed a statistically significant different pattern of expression in mild and severe asthma with each other (P=0.0001) and with healthy control (P= 0.293 and 0.001, respectively, (Figure 1B).

The fold change analysis of *IL-17A* expression showed a correlation with severity of asthma so that its expression level showed a meaningful pattern for mild and severe asthma compared to the healthy control (Figure 1C). While *IL-10* is up regulated in mild asthma patients, it decreased in severe asthma patients (Figure 1D). Nevertheless, the pattern of *IL-10* expression level did not show any correlation with the progression of asthma. Noteworthy, the down-regulation of *IL-17*A is more distinctive in all asthma samples in comparison with *IL-10* expression level (Figures 1C and D).

### Correlation analysis of IL-10 and IL-17A in mild and severe asthma groups

**Figure 2.**
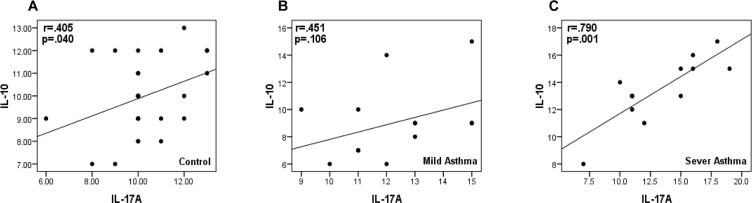
Correlation of *IL-17A*and *IL-10* expression level in normal (A), mild (B) and severe (C) cases. Patients with mild asthma showed no correlation between these genes, while there is a statistical significance correlation between expression of two target genes of *IL-17A*and *IL-10* in severe asthma patients.

To assess the possible correlation in the expression level of *IL-10* and *IL-17A* genes, the expression level of *IL-10* was compared to those of *IL-17A* gene in Pearson correlation method. There was a significant (r=0.405; p-value=0.040) positive correlation between *IL-10* and *IL-17A* expression level in normal control population (Figure 2A). In addition, to assess correlation in normal population, this parameter was calculated for patients and results showed that unlike absence of correlation in patients with mild asthma (r=0.451; p-value=0.106) (Figure 2B), there was a significant correlation (r=0.790; p-value=0.001) between *IL-10* and *IL-17A* transcription level in severe asthma (Figure 2C).

### Lack of association between IL-10 and IL-17A expression level and age

**Figure 3.**
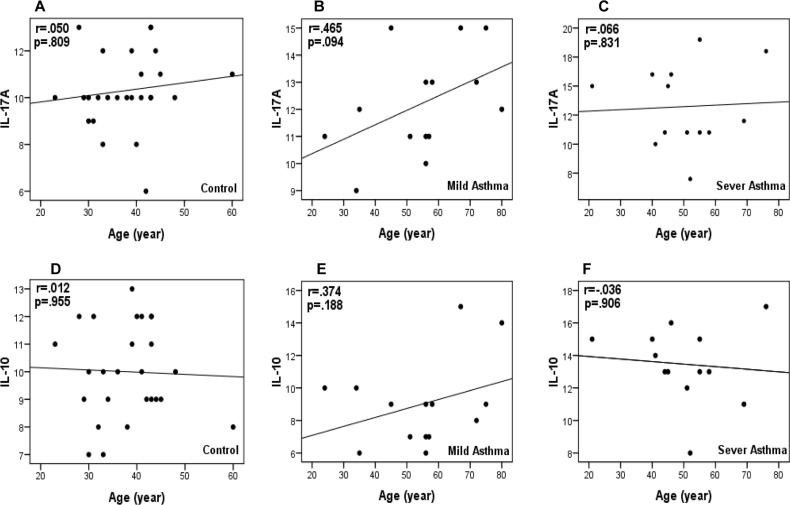
Relationship between the expression level of *IL-17A*and *IL-10* genes and ages of individuals with healthy (A and D), mild asthma (B and E) and severe asthma (C and F) situations. As shown, the scattering of expression levels (denoted as non-fill points) is not statistically significant.

To check if there was an association between expression level of *IL-10* and *IL-17A*, correlation co-efficient was calculated separately in normal, mild and severe populations. Data revealed that there was not any significant association between the expression level of these genes and age (Figures 3 A, B, C, D, E, and F).

## DISCUSSION

A growing amount of evidence suggests that different types of asthma such as the severe type could be associated with interleukins' production and accumulation (12). Of those, IL-17 may be one of the major cytokines involved in exacerbation of bronchial asthma (26). IL-17A is one of the IL-17 family members and it is considered as a pro-inflammatory cytokine, playing important role in the induction and propagation of different immunological symptoms such as alveolar inflammation (27). IL-10, as an inhibitor of cytokines from T-helper type 1 cells, is a cytokine derived from CD4+ T-helper type 2 cells (28,29). The role of IL-10 in asthma pathogenesis has not been well documented. Some evidence suggests that the production of IL-10 is enhanced in the bronchial mucosa of asthmatic subjects in comparison to non-asthmatic control subjects (30).

Although many genes (including cytokine genes) have been identified or suspected to be involved in the pathogenesis of asthma, their expression analyses were restricted to body fluids, sputum, parenchyma, airways, Bronchoalveolar Lavage (BAL) fluid and serum from patients with asthma. The expression pattern of *IL-10* and *IL-17A* not only differs from different tissues of same patient, but it differs from one population to another one. There was no reported analysis for *IL-10* and *IL-17A* expression level in peripheral blood cells of Iranian patient population. The family of IL-17 cytokines preferentially is produced from TH17 cells (31). IL-10 is an anti-inflammatory cytokine that is produced from TH2 cells and suppresses the secretion of pro-inflammatory cytokines (32). Changes in production of IL-10 and IL-17A could be consequence of several events occurring in cytokine-producing cells. It is known that IL-17 is increased in BAL fluid, sputum and blood of asthmatic patients (33). While excessive production of cytokines in lung tissue is sufficient to explain most pathogenic features of asthma, expression level of many cytokines such as IL-10 and IL-17A in blood cells needs to be investigated.

In this study, we compared the mRNA expression of *IL-10* and *IL-17A* in three distinct groups of healthy control, mild and severe asthma to investigate any relationship between these interleukins with the severity of asthma. Different groups of individuals were included in our study and the expression level of these two cytokines was compared with parameters such as the age of patients. Assessment and reporting significant and obvious expression level alteration of these genes provides the conceptual mechanisms that confer susceptibility to asthma and also would certainly simplify clinical evaluation of asthma severity detection.

Obtained result indicate that there is a significant difference in mRNA level of *IL-17A* in the individuals with mild and severe asthma compared to healthy non-asthmatic (P-value=0.006 and 0.046, respectively), but there was no difference between the individuals with mild and severe asthma (P-value=0.65). Previous studies reported increased expression level of *IL-17* in asthmatic airways, BAL fluid, lung or sputum but not in the blood cells and considered pro-inflammatory role for it (34,35). Inflammation, which is a characteristic of rheumatoid arthritis, inflammatory bowel disease and psoriasis (36), is attributed to IL-17 accumulation (37). Observed decrease of *IL-17A* expression level probably occurs because of lesions in mono-nuclear blood cells of mild and severe asthmatic individuals; these cells aberrantly expressed *IL-17* which consequently triggers production of IL-6, IL-8, Granulocyte-Colony Stimulating Factor (G-csf), CXCL1 and Macrophage Inflammatory Protein which their elevation was reported in sera of individuals with asthma (38,39). Nevertheless, the non-significant difference of *IL-17* expression level between severe and mild asthma (Figure 1A) could be explained by epigenetic events which attenuate gene expression in genome of patients with mild asthma (40). In the absence of epigenetic modifications, the lower level of *IL-17A* in the population of mild asthma may be because of low longevity of *IL-17A* mRNA (41).

IL-10 is a pluripotent cytokine with widely distribution that plays a dual role in inflammation. Decreasing IL-10 production is accompanied by the increase of pro-inflammatory cytokines' production which consequently leads to chronic inflammation, airways remodeling, airflow obstruction and lung tissue damage (29). Polymorphisms in the gene of IL-10 has been defined to play a substantial role in the inflammatory response during the onset of asthma (42,43).

Our findings showed a significant decreased level of *IL-10* in patients with severe persistent asthma compared to those with mild asthma and controls (Figures 1B and C). However, difference in expression level of *IL-10* between mild asthmatic patients and healthy individuals was statistically significant. This data suggests that because the change of *IL-*10 expression is limited to late stages of asthma, it could be useful for prognostic marker of asthma.

Since attenuated production of intracellular IL-17A and IL-10 in mononuclear cells from patients with severe asthma could not be explained by progression of asthma stages, we decided to investigate if the expression levels of *IL-10* and *IL-17A* are correlated. Correlation between these values in healthy individuals and severe patients suggests that the expression changes of *IL-10* and *IL-17A* ought to be due to common suppressive events occurring in PBMCs that finally resulted in decreased level of transcription; the events responsible for down-regulation of *IL-10* and *IL-17A* are not probably independent. This suggests that there may be interplay between the two cytokines in the pathogenesis of asthma and their correlated expression ablation contribute in asthma progression. However, lack of significant correlation between *IL-10* and *IL-17A* in mild asthma patients suggests different function of these cytokines during initiation of asthma which environment is considered as a causal role (44,45). Consistently, we found that the *IL-10* expression level is not correlated to the severity of asthma and other factors may be contributing. Further studies of larger patient cohorts and blood samples are needed to confirm and explain these findings and study of correlation between expression and the protein of target genes would be interesting.
